# Supplementary material for: Are carbon emissions trading and green financial instruments synergistic? -Comprehensive quantitative research based on content analysis
Source: PLoS One. 2024 Mar 7;19(3):e0298601. doi: 10.1371/journal.pone.0298601 (PMC10919601; doi:10.1371/journal.pone.0298601)
Supplement: S1 Data — (ZIP) [file pone.0298601.s001.zip › Data/地级市Z_F工作报告.zip!/╦─┤¿/╣π╘¬╩╨/└┤╫╘╣1⁄2╓┌║┼í╛┬φ┐╦╩2╛▌═°í┐_2016.docx]

各位代表：

现在，我代表市人民政府向大会报告工作，请予审查，并请市政协委员提出意见。

“十二五”主要成就及2015年工作回顾

“十二五”时期，是我市全面完成灾后重建任务、经济社会取得长足发展的五年，也是全市有效应对各种复杂局面、加快建设“美丽广元、幸福家园”取得重大成就的五年。五年来，我们认真贯彻落实党中央国务院、省委省政府和市委各项决策部署，在市人大和市政协的监督支持下，紧紧依靠全市人民，全面履行政府职能，攻坚克难，砥砺奋进，圆满完成“十二五”规划主要目标任务，全市呈现出经济较快增长、改革全面深化、民生持续改善、社会和谐稳定的良好局面。

五年来，我们始终保持专注发展定力，经济综合实力明显增强。地区生产总值连续跨越3个百亿台阶，五年间经济总量净增283.56亿元。全社会固定资产投资累计完成2701.73亿元，年度投资均保持在500亿元以上。社会消费品零售总额年均增长14.7%。地方一般公共预算收入年均增长19.5%。产业结构进一步优化，三次产业结构从23.8︰39.0︰37.2调整为16.5︰47.2︰36.3。工业化率从32.6%提高到40.4%，正加速迈进工业化中期阶段。建成国家级经济技术开发区1个、省级经济开发区4个。粮食总产量持续增长，新建现代农业园区47个，国家有机产品认证示范创建县2个，认定全国绿色食品原料生产基地165.6万亩。区域性商贸物流中心、旅游集散中心初具规模。万达、海博春天等一批重大商贸物流项目建成运营；建成国家5A级旅游景区1个、4A级旅游景区14个、国家级生态旅游示范区1个。县域经济实力不断提升，利州区、苍溪县经济总量分别突破200亿元、100亿元。创新驱动发展取得新进展，科技创新对经济增长贡献率达46%，高新技术产业产值增长3倍。非公有制经济占地区生产总值比重达58%。

五年来，我们全力推进基础设施改善，区位优势和承载能力大幅提升。连接西南西北、通江达海的区域性综合交通枢纽基本建成，区位优势更加凸显。广南、广陕、广甘和绕城高速建成通车，境内高速公路总里程达392公里，是“十一五”末的2.84倍。兰渝铁路广元至重庆段建成通车，西成客专加快建设。实现所有县区通高速公路和铁路。提升改造832公里国省干线公路。实现100%乡镇、92%建制村通油路或水泥路。广元机场开通北京、上海、广州、深圳、杭州五条航线。广元港红岩作业区建成试运行。苍溪乐园、青川曲河、朝天双峡湖等中型水库开工建设，建成各类水利工程7.2万余处，解决了138万农村人口安全饮水问题。建成川东北地区首个光网城市，3G网络、4G网络、广播电视、宽带进村覆盖率分别达到98%、93%、98.84%、66.96%。亭子口水利枢纽、中石化天然气净化厂、芳地坪风力发电场等项目建成投产，区域性能源供给中心初步建成。

五年来，我们强力推进城乡统筹发展，协调发展格局逐步形成。深入推进以人为核心的城镇化，梯次鲜明、布局合理的城镇发展体系基本形成。城镇化率从32.95%提高到40.83%。万源新区基本建成，完成电子路北延线、西滨道拓宽改造等一大批市政重点工程，中心城区“三横九纵”道路交通体系初步形成。15个镇入选省级“百镇建设行动”试点镇，14个镇入选国家级重点镇。建成幸福美丽新村935个、新型农村社区349个、改造农村危房8.5万户，农村公共服务设施不断完善。城乡居民收入比由3.1︰1缩小到2.64︰1。森林覆盖率达55.3%，节能减排和环保指标全面完成，城乡环境综合治理深入推进，城市污水集中处理率和城市生活垃圾处理率分别达90%、97.6%。实行定路段、定任务、定人员、定奖惩“四定”网格化城市管理，城市精细化管理水平不断提升。利州区成功创建省级可持续发展试验区。我市成为国家生态文明建设先行示范区、全省唯一国家低碳城市试点市，作为西部唯一城市获邀参加在南非德班召开的联合国世界气候大会，成功创建中国低碳生态先进城市、国家森林城市、国家卫生城市、省级环保模范城市。

五年来，我们全面深化改革扩大开放，体制机制创新成效显著。集中力量推进重点领域和关键环节改革，形成“三园一区”统筹城乡改革发展路径，供销社综合改革、农村产权“七权同确”和“1+3”新型农业经营体系等农村改革走在全省前列。简政放权、行政审批制度改革取得新成效。商事登记制度等改革有力推进。全面启动农村信用社改制组建农商银行，银行业金融机构各项存款净增418亿元，各项贷款年均增长19%，存贷款余额分别是“十一五”末的1.6倍、2.3倍；保险业保费收入实现33.3亿元，是“十一五”末的1.89倍。开放合作不断深化，积极发挥长江经济带与丝绸之路经济带、成渝经济区与关天经济区纽结点作用，深入开展川陕甘毗邻地区合作，不断加强“浙广”、“九广”合作，与乌鲁木齐、太原等地结为友好城市。成功引进世界500强企业6户和国内500强企业17户；累计引进到位市外资金1772.66亿元，是“十一五”时期的3.32倍。

五年来，我们加快发展民生社会事业，人民生活水平不断提高。财政用于民生的支出累计达568.18亿元，占一般公共预算支出65.3%，是“十一五”时期的1.4倍。减少贫困人口40.2万人，贫困发生率从26.2%下降到10.08%。城镇累计新增就业20.5万人，农村劳动力转移就业常年保持在95万人。城镇低保基本实现应保尽保，农村低保和五保供养逐年扩面提标，三项基本医保覆盖287.3万人，“十大救助制度”全面建立。改造危旧房棚户区和建设保障性住房7.6万套，向农民工提供公租房2350套。幼儿教育毛入园率、三类残疾儿童入学率、高中阶段毛入学率均实现大幅提升；义务教育均衡推进，“三免一补”、农村义务教育“营养餐”实现全覆盖；职业教育加快发展；积极引入社会力量办学，天立国际学校、万达中学建成投入使用。建成三级医院9家，数量居全省第3位；改扩建乡镇卫生院110所，新建标准化村卫生室690所，每千人常住人口拥有床位数是“十一五”末的1.48倍。环境空气质量排名全省前列，境内江河湖泊水质优良；食品药品安全得到有效保障，成为全省首个农产品质量安全监管示范市，四县三区全部纳入全省发展无公害农产品、绿色食品区域。文化体育、科普事业繁荣发展，本土特色文化精品不断涌现，成功创建中国楹联文化城市、中国散文之乡；全民科学素养全面提升；全民体育健身活动蓬勃发展；体育设施极大改善，全市大型体育场馆总数达15座，位居全省前列。提炼形成“厚德行广、坚韧自强、创新开元”的新时期广元精神，市民文明素质显著提高。

五年来，我们持续深入推进依法治市，社会大局保持和谐稳定。“法律七进”活动深入开展，“六五”普法圆满完成，累计创建全国法治示范点12个、省级法治示范点32个。公共法律服务体系逐步完善。切实加强安全生产监管，依法关闭煤矿34个、非煤矿山225个，重点行业领域安全专项整治深入推进，安全生产形势保持总体稳定。“五建五强”群众工作法和网格化服务管理模式基本形成。社会稳定风险评估制度、社情民意调查机制、多元化矛盾纠纷调处机制和应急处置机制初步建立，群体性事件和信访总量明显减少。治安防控体系逐渐完善，“平安广元”建设深入开展，社会治理能力逐步提升，全省平安建设满意度第三方测评中广元公众治安满意度连续三年位列全省第一，连续7年没有发生影响恶劣的案件、事件和重特大安全生产责任事故。成功创建无毒害市。创建无邪教危害县区成效明显。

各位代表！2015年，我们牢牢把握稳中求进的工作总基调，统筹稳增长、调结构、促改革、惠民生、防风险，较好完成了市人代会确定的目标任务，实现了“十二五”圆满收官。全年实现地区生产总值605.43亿元，增长8.6%，增幅居全省第8位；地方一般公共预算收入40.82亿元，增长16.6%，增幅居全省第4位；城乡居民人均可支配收入分别达到23628元、8939元，分别增长8.6%、10.4%，增幅均居全省第5位；居民消费价格指数上涨1.9%。

一是经济运行总体稳中趋好。面对持续加大的经济下行压力，我们认真贯彻落实国家、省稳增长安排部署，及时出台工业经济稳增长十五条、促进房地产发展三十五条等系列措施，有力促进了全市经济持续健康发展。充分发挥稳投资的关键作用。争取中央专项建设基金17.2亿元。常态化多批次开展项目集中攻坚和季度集中开工，中纺60万吨油脂加工等18个重大项目竣工，七盘关国际石材城等48个重大项目加快推进，大寨水库等30个重大项目开工建设，广元国际生态休闲养老产业园等16个重大项目前期工作有序开展。全社会固定资产投资实现583.23亿元，增长3.8%。积极拓展市场拉动消费。组织2300家企业参加国内外市场拓展“三大活动”，成功举办2015年中国·白龙湖搏鱼大赛、曾家山杯生态美食烹饪全国邀请赛、女儿节旅游商品展销会、秦巴山区年货节等活动，积极培育康养、休闲旅游、电子商务等新的消费热点，实现社会消费品零售总额296.62亿元，增长12.3%。银行业金融机构各项存款余额1135.37亿元、增长12.9%，各项贷款余额550.79亿元、增长14.1%。新三板、区域性场外交易市场挂牌企业总数达32家。

二是转型升级取得新进展。大力实施“工业转型升级三年行动计划”，设立1亿元工业发展基金，指导启明星等15户企业争取到直购电政策，落实工业企业用气指标，全力推进工业稳增长、调结构。实现工业增加值244.64亿元，增长9.7%，其中规模以上工业增加值增长10.8%。新培育亿元以上企业10户、规模以上企业50户。完成工业技改投资131亿元，淘汰落后和化解过剩产能企业70户。朝天区工业集中区成功创建省级经济开发区。实现第一产业增加值99.76亿元，增长3.7%。持续发展壮大“3+5”百亿农业产业，粮食生产实现“九连增”。新建现代农业园区7个，培育新型农业经营主体1661户。实现第三产业增加值220.14亿元，增长9.7%。剑门蜀道剑门关旅游区创建成国家5A级旅游景区，剑阁县、利州区创建成全省旅游强县，曾家山、女皇温泉创建成省级旅游度假区，成功举办剑门蜀道三国文化旅游节。全年接待游客3251.97万人次、增长17.2%，实现旅游总收入207.16亿元、增长30.53%。出台大众创业万众创新实施意见和十四条措施，新增国家高新技术企业11家。专利申请首次突破一千件。取得重大科技成果37项，获全省科技进步奖5项。实施科技成果转化项目150个，实现产值168亿元，增长5%。不断深化“厅市合作”、“市校合作”，是省内首个与清华大学签订全面合作协议的地级市。

三是改革开放不断深化。出台改革方案24项。财政改革全面深化，财政支农资金形成的经营性资产股权量化到集体经济组织成员的做法得到省委省政府充分肯定。社会投融资实现新突破，签约PPP项目10个，总投资177.16亿元。在全省率先全面推进“三证合一”登记制度改革，新登记各类企业增长17.8%。农村产权抵押融资取得历史性突破，银行间市场直接债务融资实现大幅增长。供销、教育、医疗、社会保障、公务用车等领域改革稳步推进。加强与川东北经济区、嘉陵江流域、省际地区基础设施互联互通，深化与重点地区的常态化合作，成功举办广元市（北京）项目推介会、中国西部（四川）国际投资大会广元市投资说明会暨合作项目签约仪式等重大投资促进活动。全年实施招商引资项目697个，引进到位市外资金412.95亿元，其中省外资金201.76亿元。

四是统筹城乡发展更趋协调。市县乡三级城乡规划管理机制初步建立，城乡规划体系进一步完善，完成城市总规中期评估、新型城镇化等专项规划和500个新村聚居点规划。坚持中心城市、县级城市和中心城镇梯次发展，完成城乡建设总投资165.4亿元，全市新增建成区7平方公里，城镇化率提高1.5个百分点。扎实推进小城镇建设，省级“百镇建设行动”试点镇基础设施项目基本完成，完成投资7.7亿元，就地就近吸纳转移人口1.6万人。新建幸福美丽新村211个、农村聚居点304个，改造农村危旧房2.09万户。城乡交通发展提速，广陕广巴高速公路连接线建成通车，西二环道路等重点项目加快推进；新建农村公路1100公里、路侧护栏1592公里，建成渡改人行桥9座。加快水利工程建设，上石盘电航枢纽投入运行；新建（改建）农村供水工程630处，解决了9.3万农村人口和2.8万农村学校师生饮水安全。

五是民生改善和脱贫攻坚成效显著。全面完成十项民生工程和30件民生大事，民生支出占一般公共预算支出的66.8%。组织开展首届创新创业大赛，扶持创业1.68万人，新增城镇就业4.16万人，城镇登记失业率3.89%。转移输出农村劳动力97.2万人，劳务总收入154.55亿元，新增农民工和企业家返乡创业6889人，创办经济实体4609家。深入推进“全民参保登记计划”，城镇五项社会保险累计参保159.92万人，城镇居民医保参保人员全部纳入大病保险范围，为3.04万名重度残疾人发放护理补贴2074万元。开展公益性初级应急救护培训11.07万人。开工建设各类保障性住房1.06万套，向农民工提供公共租赁住房814套。深入推进精准扶贫脱贫，全年减少贫困人口6.58万人，实现易地扶贫搬迁3174户1.22万人。扶贫小额信贷“旺苍模式”在全省推广。

六是社会事业加快发展。优先发展教育事业，第二期学前教育三年行动计划顺利推进；农村义务教育薄弱学校基本办学条件改善项目和“四均一强”工程全面实施，青川县建成全国义务教育基本均衡县，利州区顺利通过省政府义务教育均衡发展评估验收；高考本科上线连续四年稳定在万人以上；职业教育服务经济社会能力显著增强；高等教育加快发展。大力发展卫生计生事业，分级诊疗制度全域实施；公立医院改革全面推进，市、县公立医院全面取消药品加成，被列为国家城市公立医院综合改革试点；推行基层卫生院和村卫生室双签约服务，签约率达95%；荣获全国基层中医药工作先进单位。着力发展文化体育事业，市县乡村四级公共文化服务体系基本建成，群众文化活动蓬勃开展，一批文艺作品荣获国家、省级奖项；全民健身蓬勃发展，竞技体育取得新成绩，省第十三届运动会、第九届残运会、第四届特奥会筹备工作扎实推进。建成国家级地震安全示范社区4个。建成国家级科普示范社区4个、省级科普示范社区16个。审计、统计、住房公积金、测绘、气象、水文、工会、妇女儿童、青少年、老龄、残疾人、地方志、红十字会、慈善、外事侨务、港澳台、民族宗教、爱国卫生、档案等工作都取得了新成绩。

七是生态文明建设扎实推进。深入实施低碳发展和资源转化战略，大力推进节能减排，发展节能项目，淘汰落后产能，建设低碳产业园区，能源结构、产业结构、消费方式低碳化取得实质性进展。主要污染物总量减排目标任务如期完成。市城区和县级城镇空气质量优良天数比例均达到95%以上。青川县、利州区通过省级生态县验收。实施新能源示范市创建工程，持续推进“气化广元”和新能源开发利用，万元GDP综合能耗下降3%。狠抓重点流域水环境综合整治和饮用水源保护，白龙湖网箱养殖全部取缔。持续深化城乡环境综合治理，农村生活垃圾治理通过国家十部委现场核查。重点重金属污染物排放实现零增长。

八是依法治理深入推进。四级网格化社会管理服务体系不断完善，社区管理服务能力全面提升，新建新型农村社区7个。“四个三”法律七进特色做法在全省推广，成功创建全国民主法治示范村2个。“五建五强”群众工作法深入推进，服务群众平台体系进一步健全。深入开展“信访工作走基层行动”，全市信访总量下降31.9%，为近五年来信访总量最低。深化“平安广元”建设，严厉打击各类违法犯罪活动，公众安全感和群众治安满意度保持在全省前列。严格落实安全生产责任，全面推进安全生产监管体制改革，安全生产“打非治违”专项行动、道路交通安全综合整治“深化巩固年”行动取得良好成效，安全生产形势稳定向好。积极推进县区食品药品监管体制改革，加强食品药品、农产品安全监管，未发生大的食品药品安全事故。积极科学应对甘肃陇南锑泄漏污染事故，保障了广元境内嘉陵江沿岸群众用水安全。国家安全、保密、消防、人防、国防动员、民兵预备役、双拥等工作全面加强。

九是政府自身建设成效明显。加快推进政府职能转变，不断深化机构改革和行政审批制度改革，制定政府权力清单、责任清单。深化“两集中、两到位”，不断完善四级政务服务体系，受理事项办理提速90.83%。强力整治重点领域突出问题，经济发展软环境进一步优化。深化政府信息公开，推进决策、执行、管理、服务和结果公开。办理人大代表建议168件、政协委员提案328件，办结率100%，满意率98.5%。全面落实党风廉政建设责任制，创新反腐败工作体制机制，加强对权力运行的制约和监督。强化监察、审计工作。坚决纠正损害群众利益的不正之风，严肃查处“四风”和腐败问题，党风廉政建设群众满意度全省第二。

各位代表，总结过去五年的丰富实践，我们的主要经验和体会是：必须突出发展第一要务，坚持把党中央国务院、省委省政府的方针政策和决策部署与我市实际结合起来，把政策机遇转化为现实发展动力，创新发展思路，转变发展方式，推动结构调整。必须突出民本民生导向，坚持把保障和改善民生作为一切工作的出发点和落脚点，关心帮助特殊困难群体，大力推进精准扶贫脱贫，千方百计扩大就业，让人民群众共享改革发展成果。必须突出改革开放创新，坚持以改革创新思维谋划和推动发展，大胆破除制约发展的体制机制障碍，增强开放发展优势，全方位激发全社会创新创业创造潜能。必须突出筑牢法治基石，坚持以法治思维和法治方式推动发展，加快建设法治经济和法治社会，营造公开公平公正的发展环境，为改革发展稳定提供坚强保障。必须突出凝聚发展合力，坚持以人民群众的期盼为奋斗目标，以新理念引领新实践，充分调动劳动、知识、技术、管理、资本的活力，凝聚攻坚决胜的强大合力，不断开创发展新局面。

各位代表，过去的五年，科学发展、加快发展硕果累累，这是省委、省政府正确领导的结果，是市委统揽全局、科学决策的结果，是全市人民齐心协力、不懈奋斗的结果。在此，我代表市人民政府，向全市各族人民，向人大代表、政协委员、各民主党派、工商联、无党派人士、各人民团体和社会各界人士，向驻广解放军、武警官兵、政法干警和民兵预备役人员，向所有关心支持广元发展的朋友们，表示诚挚的谢意!

在充分肯定成绩的同时，我们也清醒地认识到，我市发展不足、发展滞后的基本市情没有改变，推动广元发展仍然面临诸多困难和挑战，主要是：主导产业支撑经济增长的后劲不足，自主创新能力不强，转型发展尚处于起步阶段，促进投资和消费持续较快增长的难度加大，新型城镇化可持续发展乏力，县域经济实力不强，脱贫攻坚任务繁重，城乡居民收入水平偏低，人才支撑作用不强，政府系统工作人员观念、作风、能力等方面还存在许多不足。我们将进一步强化忧患意识和服务意识，正视矛盾，直面问题，在今后工作中努力加以解决。

“十三五”发展的主要考虑

“十三五”时期是全面建成小康社会的决胜阶段。根据国家、省“十三五”规划和市委六届十一次全会精神，市政府组织编制了《广元市国民经济和社会发展第十三个五年规划纲要（草案）》，已印发大会审查。这里，我从四个方面作一简要说明。

关于发展形势。规划纲要的编制，深刻把握了发展形势和我市的阶段性特征。今后五年，是广元科学发展、转型发展、加快发展的重要战略机遇期。我国经济发展长期向好的基本面不会改变，国家、省多重战略和政策叠加，将会释放更多红利。但战略机遇期内涵已发生深刻变化，经济增速、工业品价格、实体企业盈利、财政收入增幅下降，经济风险发生概率上升，加之自身长期积累的结构性矛盾突出，发展仍面临诸多严峻挑战。经过多年的艰苦努力，我市经济发展在新常态下进入新的阶段，呈现以下主要特征：一是经济发展转型升级急需突破。虽然全市经济总量突破600亿元，但总量仍然很小，因此，做大总量与提档升级是长期面临的双重任务，必须坚持在做大总量中转方式，在扩大增量中调结构，做到“总量做大、结构趋优、转型加快、创新增强、质量向好”。二是推进发展的新旧动力转换加速。经济发展正由主要依靠投资拉动向依靠投资和消费均衡拉动转变，由主要依靠工业增长向依靠工业和现代服务业同步增长转变，由主要依靠要素驱动向依靠要素和创新双轮驱动转变。三是“四化”融合联动发展加快。我市的产业结构、城乡结构、社会结构已发生深刻变化，正在实现以农村社会为主向城镇社会为主的历史性跨越，工业化、信息化、城镇化、农业现代化正在加快融合发展。四是对外开放合作全面提速。广元处于成渝西昆菱形经济圈的重要节点，是丝绸之路经济带与长江经济带、成渝经济区与关天经济区的纽结点。随着“一枢纽三中心”的基本建成和“高铁时代”即将到来，我市开放合作面临重大机遇。五是经济底部基础进一步夯实跃升。我市“一带一廊两区”空间和产业布局基本形成，市域特色城镇群竞相发展，县域经济不断壮大，底部基础进一步夯实，正在进入快速跃升的新阶段。

关于发展原则。立足我市经济社会发展的阶段性特征，深入贯彻新的发展理念，坚持和遵循以下原则：一是始终坚持科学发展、转型发展、加快发展。把经济建设作为兴市之要，着力加快发展转型升级，实现更高质量、更有效率、更加公平、更可持续的发展。二是始终坚持生态立市、工业强市、文旅兴市、统筹发展。推动经济与环境、人与自然协调发展，做大工业经济规模，深入推进文旅融合发展，统筹城乡发展、经济社会发展、区域协调发展，构建全面、协调、可持续发展新格局。三是始终坚持改革创新、开放合作。加快完善以经济体制为重点的各项体制机制，推进以科技创新为核心的全面创新，扩大全方位开放合作，提高经济外向度和社会开放水平，构建发展新优势。四是始终坚持尚法守制、依法治理。全面实施依法治市方略，加快形成法治经济和法治社会，以法治思维和法治方式推动发展，为经济社会发展创造良好的法治环境。五是始终坚持共建共享、发展为民。把改善和提升人民生活质量、促进人的全面发展作为发展的出发点和落脚点，坚持脱贫攻坚和全面小康共同推进，使全市人民共享改革发展成果。六是始终坚持党的领导。改进工作机制和方式方法，加强各级领导班子和干部队伍建设，提高抓经济发展和脱贫攻坚的能力，持续改进作风和推进反腐倡廉，为经济社会持续健康发展提供坚强政治保障。

关于发展目标。着眼实现同步全面建成小康社会、加快建设“美丽广元、幸福家园”的总体任务，提出以下具体目标：一是经济增长速度高于全国全省平均水平。地区生产总值年均增长8%以上；产业结构进一步优化，科技创新能力明显增强，转型发展迈出实质性步伐。全面建成区域性综合交通枢纽、商贸物流中心、旅游集散中心和能源供给中心。二是人民生活水平和质量明显提高。城乡居民收入保持较快增长，收入差距明显缩小，贫困县区、贫困村全部摘帽，全面消除绝对贫困；就业创业稳步扩大；公共服务体系更加健全。三是市民素质和社会文明程度显著提升。基本形成尚法守制、公平正义、诚信文明、安定有序的依法治市新格局。四是国家生态文明先行示范区和生态经济示范区基本建成。生产和生活方式低碳化绿色化取得显著成效，全面完成国家、省下达的各项环保指标任务，资源节约型和环境友好型社会建设取得更大进展。五是全面深化改革实现重大突破。市场在资源配置中的决定性作用得到充分发挥，内外联动、务实高效的开放合作新格局基本形成，法治政府基本建成。

关于发展重点。围绕新的发展理念，坚定走符合广元实际的发展之路，主要突出以下发展重点:一是坚持创新发展。围绕解决发展动力问题，重点在实施创新驱动战略、完善创新发展新体制、促进产业转型发展、建设现代基础设施体系、推动信息化与经济社会融合发展等方面着力，加快培育经济增长新动能。二是坚持协调发展。围绕解决发展不平衡问题，重点在实施大项目促大发展战略、推进新型城镇化、加快建设幸福美丽新村、推进城乡统筹、夯实县域经济综合实力、推动物质文明和精神文明协调发展等方面着力，加快形成平衡发展新格局。三是坚持绿色发展。围绕解决人与自然和谐问题，重点在加快建设主体功能区、大力实施低碳发展战略、深入实施资源转化战略、强化生态保护和建设、构建嘉陵江上游生态屏障、提高防灾减灾能力等方面着力，加快建设生态文明新广元。四是坚持开放发展。围绕解决内外联动问题，重点在深化全方位开放合作、提升开放型经济新水平等方面着力，加快形成合作共赢新局面。五是坚持共享发展。围绕解决社会公平正义问题，重点在大力实施脱贫攻坚、加快社会事业全面发展、推进依法治市和提升社会治理能力等方面着力，顺应人民美好生活新期待。

2016年主要目标任务和重点工作

今年政府工作的总体思路是：全面落实党的十八大和十八届三中、四中、五中全会、省委十届七次全会精神，以邓小平理论、“三个代表”重要思想、科学发展观为指导，深入贯彻习近平总书记系列重要讲话精神，按照“五位一体”总体布局和“四个全面”战略布局，牢固树立和贯彻落实“四个意识”和“五大发展理念”，主动适应和把握引领经济发展新常态，按照市委六届十一次全会和市委经济工作城市工作脱贫攻坚工作会议部署，始终保持科学发展、转型发展、加快发展战略定力，坚持稳中求进工作总基调，坚持生态立市、工业强市、文旅兴市、统筹发展总体思路，坚持稳增长、调结构、促改革、惠民生、防风险，大力实施“五大发展战略”，加强供给侧结构性改革，提高供给体系质量和效率，提高投资有效性，改造提升传统比较优势，加快培育新的发展动能，促进发展成果共享，推动“十三五”发展实现高点开局。

主要预期目标建议：地区生产总值增长8%；规模以上工业增加值增长9%；全社会固定资产投资增长5.5%；社会消费品零售总额增长11%；地方一般公共预算收入增长8%；城乡居民人均可支配收入分别增长9%、10%；居民消费价格指数涨幅控制在3%以内；城镇登记失业率控制在4%以内；城镇化率提高1.4个百分点；森林覆盖率达到55.5%；全面完成节能减排目标任务。

为实现上述目标，今年重点抓好以下工作：

一、增强投资消费拉动力，全力稳定经济增长

以全省“项目年”为契机，全力抓好项目投资，完成全社会固定资产投资615亿元。全面清理并研究出台系列稳增长政策措施，打好政策组合拳。着力优化投资结构，抓好基础设施、民生领域投资。突出产业投资特别是工业投资，力争完成工业投资195亿元。强化项目要素保障，继续实行重大项目“四个一”推进机制和市领导联系重点项目机制，集中力量抓好56个省级重点项目、100个市级重点项目。扎实开展季度项目集中开工活动，尽快开工广（元）平（武）高速等重大项目。加快完善境内高速公路配套对接，积极争取绵广高速复线绵阳至陕西段立项。精准对接国家、省支持政策，抓好项目储备，确保项目滚动储备1万亿元以上。激发民间投资活力，吸引更多民间资本参与重点项目建设。

积极扩大有效消费需求。加快推进消费领域改革，引导企业根据消费升级需求调整产品结构，增加中高端、多元化的服务供给。积极引导群众改变消费观念、转变消费方式。深入实施“流通效率提升”工程，大力开展“放心舒心消费城市”创建活动，着力拓展信息、绿色、住房、旅游休闲、教育文体、康养家政等消费领域，加快培育新的消费热点。加大“广元造”产品扶持和销售力度，继续开展好市场拓展“三大活动”，办好秦巴山区年货节等地方性展会，积极开拓西北、华北、东北地区市场。

二、着力推进产业转型升级，持续优化经济结构

大力实施工业强市战略。坚持需求侧牵引和供给侧优化并举、稳增长与调结构相平衡、培育新兴产业与改造传统产业并进、存量扩张与开放合作并重的思路，全力推动工业经济平稳增长和提质增效。围绕“5+2+1”产业体系，继续实施“工业转型升级三年行动计划”和工业强基工程，开展质量品牌和质量对标提升行动，探索设立战略性新兴产业发展基金，重点培育新材料、新能源、生物医药等高端成长型、战略性新兴产业发展。推进“互联网+制造业”创新发展，促进信息化工业化深度融合。推进传统优势产业转型升级，帮助金属、煤炭等产业逐步走出困境。狠抓工业重点项目建设，推动剑兴2亿安时动力锂离子电池等项目及早竣工投产。支持中小微企业“专精特新”发展，培育新进规模以上企业40户。加强园区配套能力建设，完善市场化建园模式。加快发展建筑业，力争实现总产值130亿元。

加快发展现代服务业。积极推动商业中心、专业市场、特色街区提档升级，加快推进下西商贸圈建设，着力推进红星美凯龙城市综合体等重点商贸项目，整合完善建材、农产品等专业市场。推进社区和农村商业网点建设。推动物流业集聚发展，加快“一港五园区”和卓新等重点物流项目建设。深入实施电子商务发展三年行动计划，大力推进广元电子商务园区建设，加快区域内农村邮政、快递网络布局，完善提升工业品下乡、农产品进城和农村综合服务。抓好青川电子商务进农村综合示范县建设，积极推进苍溪申报创建。突破发展现代旅游业。创建全国旅游标准化示范城市。尽快启动剑门关文化旅游产业园等项目，启动创建唐家河国家级旅游度假区、米仓山国家5A级旅游景区，启动“两湖”新兴旅游区建设。不断丰富旅游产品、旅游商品。大力推进剑阁和青川国家级全域旅游示范区建设、苍溪省级乡村旅游扶贫示范县创建。办好中国（广元）女儿节、剑门蜀道三国文化旅游节。力争实现旅游接待人数3700万人次、旅游总收入260亿元、门票收入2.5亿元。

加快发展现代农业。加快推进国家现代农业示范区、全省特色农业强市建设。做大做强“3+5”百亿农业产业，新增五大特色产业基地10万亩。大力发展现代畜牧业，突破性发展肉牛羊和生态渔业，推进剑门关土鸡产销体系建设。扎实推进“三大建设”，严格划定和保护永久基本农田，新建高标准农田8万亩，新建现代农业园区7个、提升7个，新建和整治渠系500公里。持续推进绿色农业发展，稳步发展有机农业。加大农产品品牌整合力度，做强做响“7+3”优势特色品牌。加快培育新型农业经营主体，引进和培育销售收入过亿元的龙头企业7家，新培育家庭农场300个、规范农民合作社100个、社会化服务超市100家。大力发展休闲观光农业、道地中药材综合开发利用、农产品仓储物流等新产业新业态。加强劳务品牌建设，全年转移输出劳动力保持在95万人以上，实现劳务收入155亿元。大力推广使用农业机械，提升农业现代化装备水平。做好第三次全国农业普查。

三、加快改革创新步伐，培育发展新动能新活力

创新政府投资管理方式和投融资机制，完善分行业的政府和社会资本合作管理制度，推动建立“1+X”政策制度体系。进一步防范和化解地方政府性债务风险。积极推进国企劳动、人事、分配三项制度改革，稳妥推进混合所有制改革。健全完善民营经济发展政策体系，着力推进企业转型升级。推进财税体制调整、营改增、基金转列等改革措施落地。加快建立健全现代农村产权制度，总体完成“七权同确”，完善农村产权交易流转体系，增加农民财产性收入。深化供销社综合改革，扎实推进财政支农资金股权量化和苍溪县农村土地承包经营权抵押贷款试点，稳妥推进农村土地流转收益保证贷款、林权抵押贷款，稳步扩大农村资金互助社试点范围。加快推进全市不动产统一登记改革。完成利州、昭化、朝天、青川、剑阁农村信用社改制组建农商银行。完成公务用车改革。深化医药卫生体制改革，积极推进城市公立医院综合改革国家级试点。全面完成乡镇计生服务站与卫生院整合。深化教育综合改革，推进文化事业单位法人治理结构改革。深入推进城市执法体制改革。

认真落实四川省全面创新改革试验总体方案和年度计划，积极对接成德绵协同创新发展，加快构建创新平台共建、资源共享、政策共用的新体制新机制。推进大众创业、万众创新，激活多层次、多人群创新创业，积极推进科技创新服务中心和科技孵化器建设。实施产业技术创新行动，推行科技型企业梯次培育和创新平台升级及退出机制，推进081机电产业园申创省级高新技术产业化基地。加强重点实验室、工程实验室、工程技术研究中心等科技创新平台建设，深化市校合作、厅市会商，推行成果定制，实施科技成果转化项目140个，实现产值160亿元。创建国家级农业科技示范园区和创新驱动助力工程省级示范点。推动科技与金融融合和银会、银站合作，做好科学技术普及、科普基地建设和科技扶贫工作。大力实施“海智计划”，加大人才引进力度，积极开展高层次人才引进工作。推动广元经济技术开发区申创省级百亿知识产权示范园区。

四、抓好结构性改革重点任务，着力解决经济发展突出问题

开展降成本增效益专项行动，对接国家降低企业成本总体方案，认真落实减税降费政策。进一步降低政府性收费标准，强化涉企收费目录清单管理，减轻企业制度性交易成本。积极协调争取企业直购电、丰水期富余电量消纳等优惠政策，降低重点用能企业生产要素成本。深化物流运输价格形成机制研究，尽可能降低运力成本。认真贯彻落实金融支持工业稳增长调结构增效益的政策措施，深入实施小微企业金融服务提升工程、企业上市和挂牌融资行动计划，推进利率市场化，清理银行业金融机构不合理收费，降低融资成本，努力解决实体经济资金困难。

积极淘汰落后产能，化解过剩产能。针对煤炭等行业，分业施策，支持困难行业去产能、去库存。重点抓好特种铝合金型材、5万吨稀土铝合金电缆、安驭铝合金二期100万只汽车轮毂等电解铝配套项目建设，引导电解铝、水泥生产企业实施节能技术改造。完成技改投资135亿元。支持优势企业兼并重组，按照多重组、少破产要求处置“僵尸企业”，同步做好职工安置工作。

着力推进房地产去库存。继续实施促进房地产发展三十五条措施，实现商品房销售面积增长10%。大力发展住房租赁，更好满足新市民住房需求。进一步落实房地产新政，推动棚改安置房、公租房和租赁市场与存量商品房市场有效衔接。积极引导房地产企业从单一依靠住宅开发向兼顾商业、旅游、康养和城市综合体转型，促进房地产市场良性发展。

五、大力开展投资促进，全方位推进开放合作

切实加大招商引资力度。立足生态和区位交通优势，突出特色和优势资源，精心储备、包装一批项目。坚持招大引强、好中选优，以世界500强、中国500强、央企、上市公司和国内知名民营企业、行业领军企业为重点，引进一批龙头型企业，重点抓好川能投天然气精细化工产业转移等项目。积极参加中外知名企业四川行、西博会、科博会、农博会等重大投资促进活动和展会，力争新签约项目合同资金500亿元以上，引进到位市外资金450亿元以上。健全完善招商引资工作体系、政策体系、管理机制、协调服务机制和目标考核机制，提升招商引资实效。

立足长江经济带与丝绸之路经济带纽结点地位，主动在两带联动中寻找发展空间，推进涉外投资、经贸等合作。巩固、密切南向与成渝经济区以至整个西南地区经济联系，加大北向对西北、华北、东北地区开放力度，扩大与长三角、珠三角、环渤海地区的交流合作，积极承接产业转移。多渠道开拓航线，不断提高对外航空通达能力。深化“浙广”、“九广”合作，拓展合作领域和层次。以广元经济技术开发区为重点，积极培育外向型产业示范园区和出口基地。加强与乌鲁木齐等口岸城市合作，推动特优农副产品边境贸易出口。推广苍溪县国家级出口猕猴桃质量安全示范区经验，推动更多优质农产品创新基地验放、出口直放。

六、加大城乡统筹力度，推进全域协调平衡发展

持续推进城乡规划、产业发展、基础设施建设、基本公共服务和社会治理“五大统筹”，加快形成以工促农、以城带乡、工农互惠、城乡一体的新型城乡关系。继续推进“三园一区”建设，巩固城乡一体化发展有效载体。加快三江新区建设。统筹推进城乡基本公共服务均等化，健全便民利民服务设施和农村基础设施投入长效机制，开展村级公共服务标准化试点，推动城镇公共服务向农村延伸。健全保障农业转移人口平等就业、接受教育、参加社保的政策环境。构建新型城镇体系。优化发展“一带一廊两区”城乡空间布局。完善城市交通体系，加强三江新区骨干路网、断头路改造，加快建设中心城区西二环等项目。启动城市地下综合管廊和海绵城市前期工作，改造提升城镇地下管网，加快推进市城区第二水源建设。大力实施城镇安居工程，开工改造危旧房棚户区3994套（户），基本完成危旧房棚户区改造、公租房建设3058套（户），完成危旧房棚户区改造、保障性住房建设5719套（户），实施棚改货币化安置1721套（户），向农民工定向供应公租房718套（户），发放租赁补贴6916户。试点PPP模式建设管理公租房。认真实施利州区赤化镇省级统筹城乡综合示范项目，启动市级综合示范镇建设。积极开展科普智慧城镇和社区建设。以国家、省、市重点镇为重点，加快建设一批以发展商贸、工业、农业和旅游业等为主的特色小城镇。继续推进户籍制度改革。大力提升城市文化品位，丰富城市文化内涵。

加快幸福美丽新村建设。新建幸福美丽新村250个、新村聚居点300个、农村廉租房1135户、新农村综合体7个。抓好旺苍、青川、利州省级幸福美丽新村示范县和其他县区市级示范片建设。狠抓一村一品，推动产业向新村、向园区集中，推进优势特色产业连片成带发展。加快农村新型社区建设。加强传统文化村落、古民居、乡土特色建筑保护。新、改、扩建农房1.5万户。新建农村公路1100公里，完成渡改桥16座，新建汽车客运站5个。实施农村饮水安全提质增效工程，覆盖人口10万人。

七、全力推进脱贫攻坚，加快补齐全面建成小康社会短板

坚持把脱贫攻坚作为头等大事和第一民生工程，坚持以脱贫攻坚统揽经济社会发展全局。大力实施“4456”工作方略，打好“3+12”政策组合拳，扎实推进精准扶贫脱贫，全年实现148个贫困村、6.1万贫困人口脱贫。动态管理建档立卡贫困人口，完善建档立卡数据库。大力实施“五个一批”脱贫行动计划和12个扶贫专项方案年度计划。加强扶贫资金统筹，坚持捆绑使用、定向安排、精准投放。全力配合中央国家机关、省级单位在广元开展定点扶贫工作。全面落实“六个一”帮扶机制，严格驻村帮扶工作力量管理考评。强化脱贫攻坚责任机制，压实各级各部门扶贫攻坚责任。强化县级统筹管理，落实扶贫目标、任务、资金、权责“四到县”制度，加强常态化督促、检查、指导，引入第三方监督评估，加强脱贫成效考核。

八、更好地保障和改善民生，不断增进人民福祉

集中财力办好民生实事，全市一般公共预算民生支出比重稳定在65%左右。继续实施十项民生工程，办好30件民生大事。实施更加积极的就业政策，实施高校毕业生就业促进和创业引领计划，鼓励支持农民工和企业家返乡创业。全年实现城镇新增就业3.2万人。

大力发展社会事业。提升普通高中办学质量，深入推进义务教育“四均一强”均衡发展，完成第二期学前教育三年行动计划，推进现代职业教育产教深度融合，实施好职教“雨露计划”，创办四川健康职业技术学院。支持和鼓励民办教育发展。建立社会保障待遇正常调整机制，稳步推进机关事业单位养老保险制度改革。完善覆盖城乡的社会保障体系，实现大病保险全覆盖，扩大异地就医联网结算医院范围。健全特殊困难群体社会救助体系，加快发展慈善事业。推进“健康广元”建设，健全全民医保体系，加快推进分级诊疗、医养结合，健全药品供应保障机制。推进中医药事业创新发展。加大传染病、地方病、职业病综合防治。加强妇幼卫生能力建设。关心关爱农村留守儿童和孤儿。落实全面两孩政策。大力发展文化体育事业。倡导全民阅读，建设书香社会，加快推进基层综合性文化服务中心和广元科技馆建设，完成“中华诗词之市”创建。加强文化遗产保护和传承。广泛开展全民健身活动，加紧筹备省第十三届运动会、第九届残运会、第四届特奥会。深化群众性精神文明创建活动，全力做好第四届省文明城市创建迎检工作。进一步做好民族宗教、住房公积金、红十字、地方志、爱国卫生、档案、对台等工作。

九、着力推进绿色发展，加快建设生态文明新广元

加强生态环境保护和治理。大力实施天然林保护、新一轮退耕还林、林业有害生物防治等重点工程，营林造林30万亩。加强湿地公园、湿地保护小区的规划和建设，实现城市绿化精细化管理，启动植物园建设，深入推进国家园林城市创建。全面完成国家、省主要污染物总量减排任务。深入实施大气污染防治，加大颗粒物污染管控力度，确保可吸入颗粒物（PM10）浓度持续下降。强化城镇生活污水处理厂建设和运营，继续开展“两江”、“两湖”流域污染综合治理，强化饮用水水源和良好水体保护，加快城市黑臭水体整治，全面整治市城区水域污染源。加强土壤、固体废物、重金属污染防治，建立废旧农膜收集处理体系。深入推进城乡环境综合治理“五大工程”，巩固提升国家卫生城市创建成果。加强环境监测和执法监管，推行网格化监管，加大执法惩戒力度。

加快推进国家生态文明先行示范区和生态经济示范区建设。积极推广循环工业发展模式，引导企业加快低碳技术改造。推广测土配方施肥等低碳农业技术，发展绿色优质特色农产品。加大新能源开发利用，发展农村沼气集中供气。推进国家低碳城市试点，大力培育低碳文化，积极创建低碳社会。加快推进国家新能源示范市建设。

十、加强和创新社会治理，维护社会和谐稳定

深入推进依法治市，启动实施“七五”普法，深入开展“法律七进”和法治示范创建活动，加强法治文化建设。推进法律服务行业规范化、专业化、品牌化建设，依法做好法律援助工作，着力构建公共法律服务体系。健全基层依法治理体系和网格化服务管理体系，深入推行“五建五强”群众工作法，加强社区、社会组织、社会工作者“三社联动”。扎实开展信访“三无”创建活动，落实领导干部接访、下访、包案化解信访问题机制。引导群众依法行使各项权利，加强人民调解组织建设，完善矛盾纠纷多元化解机制。推动社会稳定风险评估全覆盖。

深入推进“平安广元”建设。强化反恐防暴、反分裂和防邪反邪，建立健全社会治安防控体系。推进“雪亮”工程建设，加强社会治安重点地区集中整治。依法严厉打击各类违法犯罪活动。压实安全生产责任，强化重大行业领域安全专项整治，扎实开展道路交通安全综合整治，强化消防安全监管，加强安全生产监管监察能力和防灾减灾能力建设，努力减少一般事故、遏制较大事故、杜绝重特大事故。加强国防动员和后备力量建设，做好双拥、人民防空等工作。严厉打击非法集资和逃废银行债务行为，依法规范投融资理财机构发展，维护银行资产安全和区域金融稳定。做好防震、气象、测绘、地质灾害防治等工作，有效防范和应对重大自然灾害。加强市场监管，维护良好市场秩序。实施最严格的食品药品安全监管，从严查处各种违法违规行为。

十一、聚焦转变职能和提高效能，切实加强政府自身建设

加强法治政府建设。厘清政府与市场、社会边界，完善政府权力清单、责任清单和负面清单。健全落实重大行政决策终身责任追究制度及责任倒查机制，推进依法科学民主决策。深化行政执法体制改革，推进综合执法。进一步提升规范性文件质量。加强行政权力运行监督制约，认真落实“三同”机制。认真执行人大及其常委会的决议、决定，主动接受人大工作监督、法律监督和政协民主监督，加强行政复议和行政应诉工作。

加强服务政府建设。继续清理行政审批事项，规范行政审批行为，简化优化公共服务流程，全面实施商事登记“三证合一、一照一码”，推动省、市、县三级联审体系建设，推进行政审批一体化运行。推行“互联网+政务服务”，全面深化政务公开，及时回应社会关切。加强政务督查、效能监督和发展环境治理，坚决纠正不作为、乱作为，治理懒政、怠政，惩处失职、渎职。

加强廉洁政府建设。巩固提升群众路线教育实践活动成果，践行“三严三实”，持续纠正“四风”，加强政风行风和效能建设，切实解决损害群众利益的突出问题。严格落实从严治政各项规定及党风廉政建设“两个责任”，把廉洁从政贯穿政府工作各个环节。发挥行政监察、审计监督和社会监督作用，努力让人民群众从政府建设和工作中感受到清风正气。

各位代表！新常态赋予新使命，广元发展时不我待。让我们更加紧密地团结在以习近平同志为总书记的党中央周围，在省委、省政府和市委的坚强领导下，紧紧依靠全市人民，开拓创新，拼搏实干，为全面建成小康社会、加快建设“美丽广元、幸福家园”而努力奋斗！
